# Mass Spectrometry Imaging for Single-Cell or Subcellular Lipidomics: A Review of Recent Advancements and Future Development

**DOI:** 10.3390/molecules28062712

**Published:** 2023-03-17

**Authors:** Dan Li, Zheng Ouyang, Xiaoxiao Ma

**Affiliations:** Department of Precision Instruments, Tsinghua University, Beijing 100084, China

**Keywords:** mass spectrometry imaging, single-cell, organelle, lipidomics, data analysis, matrix-assisted laser desorption ionization (MALDI), secondary ion mass spectrometry (SIMS), multimodal imaging, deep learning

## Abstract

Mass Spectrometry Imaging (MSI) has emerged as a powerful imaging technique for the analysis of biological samples, providing valuable insights into the spatial distribution and structural characterization of lipids. The advancements in high-resolution MSI have made it an indispensable tool for single-cell or subcellular lipidomics. By preserving both intracellular and intercellular information, MSI enables a comprehensive analysis of lipidomics in individual cells and organelles. This enables researchers to delve deeper into the diversity of lipids within cells and to understand the role of lipids in shaping cell behavior. In this review, we aim to provide a comprehensive overview of recent advancements and future prospects of MSI for cellular/subcellular lipidomics. By keeping abreast of the cutting-edge studies in this field, we will continue to push the boundaries of the understanding of lipid metabolism and the impact of lipids on cellular behavior.

## 1. Introduction

The significance of cellular biology has skyrocketed in the realm of biological sciences, particularly when it comes to analyzing cellular processes at a single-cell level, such as determining cell fate, aging, differentiation, and proliferation [1]. Cells and organelles are the fundamental units of life and play a crucial role in the functioning of biological systems. A comprehensive understanding of the molecular and cellular components of these systems is critical to advancing our knowledge of biology and medicine. Lipidomics is an area of research that concentrates on the examination of lipids, a heterogeneous collection of biomolecules that have a pivotal impact on numerous biological functions, including energy storage [2,3], membrane structure and function [4,5,6], and signaling [7,8]. Therefore, understanding the lipidomics of individual cells can give us important information about cell function and cellular signaling pathways. The single-cell approaches, e.g., fluorescence-activated cell sorting (FACS), single-cell sequencing, and mass cytometry, have been widely used. For the majority of cellular analyses, cells are typically cultured within Petri dishes or as suspensions prepared from tissue samples and isolated before being sent into instruments. However, isolating a single cell requires precise technical skills and equipment, and any mistake in the process can lead to contamination or loss of cells. In addition, the process of single-cell isolation is often low-yielding, making it challenging to study large populations of cells. The inter-cell analysis is also hindered during isolation, since the spatial distribution of the cells in the natural state is lost, especially when cells are obtained from tissues and re-cultured. Hence, image-based cell profiling has emerged as a high-throughput strategy for intracellular and intercellular analysis, enabling the quantification of phenotypic variations within diverse cell populations, which are among the various techniques used for exploring omics.

Mass spectrometry imaging (MSI) has been developed as a powerful imaging tool for analyzing biological samples at the molecular level [9]. It allows for the simultaneous measurement of multiple molecular species within cells and organelles. For cellular analysis, MSI could provide the two-dimensional or three-dimensional molecule information of cells and organelles, such as dynamic profiles of secondary ion mass spectrometry (SIMS) [10]. Moreover, with the ability to accurately identify and analyze the lipid structures of individual cells, MSI is providing new information about the complexities of cellular signaling and the regulation of cellular functions [11,12]. Despite recent advancements in MSI, MSI for cellular/subcellular lipidomics has a long way to go to become mature. The primary challenge in mass spectrometry imaging is the collection of spatial lipid information at the cellular scale (i.e., the high spatial resolution), in addition to the processing of data to enable subsequent single-cell lipidomics analysis. This review provides an overview of the recent advancements and future prospects of MSI for cellular/subcellular lipidomics.

## 2. Single-Cell and Single-Organelle Lipidomics

Single-cell lipidomics is a rapidly growing field that aims to study the lipid composition of individual cells. The significance of single-cell lipidomics lies in its ability to shed light on the biochemical processes occurring within cells and the vital role played by lipids in these processes. In addition to its fundamental importance, single-cell lipidomics also has numerous applications in the study of diseases and pathological conditions. For instance, lipid peroxidation, a chemical reaction that occurs when lipids in the cell membrane are oxidized by free radicals, leading to the formation of highly reactive and potentially harmful compounds known as lipid peroxidation products, has been implicated in several diseases and conditions, including neurodegenerative diseases, cardiovascular disease, cancer, and aging [13,14]. Furthermore, the lipid component has an impact on many human diseases, such as Alzheimer’s and cancer. Understanding the mechanisms and consequences of lipid peroxidation is therefore a critical area of research, with potential implications with regard to the prevention and treatment of various diseases. This knowledge could aid in the development of new therapies and drugs for these conditions. Furthermore, it has been shown that there is significant heterogeneity in cell-to-cell lipids [15]. Different cell types, subcellular structures, and physiological states can exhibit different lipid compositions and distributions which have significant impacts on the normal functions of cells and the development of diseases. In-depth studies of this heterogeneity may provide new insights and strategies for preventing and treating relevant diseases. Additionally, investigating cell-to-cell lipid heterogeneity can aid in the discovery of new drugs and therapeutic strategies targeting the lipid metabolism. Hence, single-cell lipidomics offer a more comprehensive understanding of the heterogeneity of cells within a tissue, while traditional lipidomics methods analyze the lipid composition of a cell population. By analyzing the lipid composition of individual cells, researchers can gain a deeper understanding of the diversity of metabolism within a tissue and the role of lipids in regulating cell behavior.

**Figure 1:**
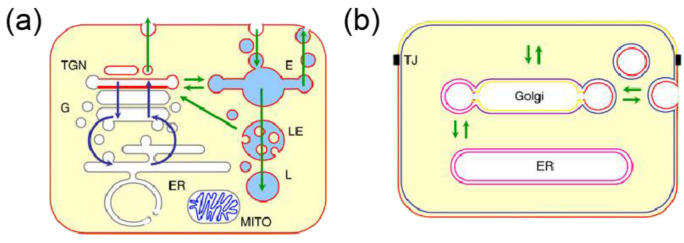
(**a**) Lipid organization in animal cells (G for Golgi, TGN for trans Golgi network, MITO for mitochondria) [19]. The glycerophospholipids are denoted by the gray color, sphingomyelin by the red color, and cardiolipids by the blue color. (**b**) Lipid sorting by lateral segregation [19].

Lipids also play a key role in the functioning of organelles, which are subcellular structures that perform specific functions within the cell. The main organelles found in these cells include the mitochondria, nucleus, Golgi apparatus, endosomes (E), endoplasmic reticulum, lysosomes (LE), and peroxisomes. The study of organelle lipidomics, which involves the analysis and characterization of lipids in specific organelles, offers numerous benefits compared to examining the entire cell. The relationship between the distribution of lipids in the organelles of animal cells are shown in Figure 1. Organelles exhibit distinct lipid compositions and profiles that are directly linked to their functions. For instance, the endoplasmic reticulum (ER) is a vital organelle that regulates lipid metabolism and is the site of lipid synthesis and modification, including phospholipids, sphingolipids, and cholesterol. By examining the lipid composition of the ER, researchers can gain a deeper understanding of the regulation of lipid synthesis and trafficking, as well as the impact of various metabolic and environmental stressors on ER function [16,17]. In the Golgi, lipids undergo various chemical modifications, such as acylation, glycosylation, and phosphorylation, which alter their properties and functionality. Mitochondria also play a crucial role in lipid metabolism and contain key enzymes involved in the oxidative degradation of fatty acids, which provides energy for the cell. Furthermore, mitochondria are the primary site of oxidative stress and lipid peroxidation, making the study of organelle lipidomics particularly relevant to understanding cellular metabolism and the associated pathologies. Additionally, researchers can gain insights into the functions of specific lipids in cellular transport and signaling by analyzing the lipid profiles of vesicles [18].

## 3. Single-Cell and Single-Organelle MSI

### 3.1. Cultured-Based and Tissue-Based Samples

**Figure 2:**
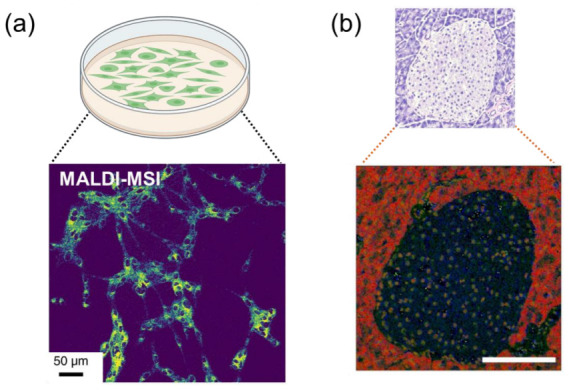
(**a**) Cultured-based samples. The MALDI MSI image of Vero B cells is from Ref [22] (**b**) Tissue-based sample. The H&E staining and SIMS-MSI images of the mouse pancreas are from Ref [23]. Scale bar, 100 μm.

For single-cell/organelles MSI, the cells and organelles used in an experiment are mainly from cell cultures and tissues (Figure 2). Despite the same aim to visualize individual cells, they are designed to cater to different requirements and present distinct advantages and limitations. For cultured cells, the implementation of cellular MSI requires several crucial steps of sample preparation, such as cell plating, washing and drying, and fixation, which may result in the rupture of cells and the spreading of cytosolic contents under adverse conditions. The optimization of sample preparations could improve the performance of single-cell/organelle MSI [20]. Despite this challenge, the controllable dispersion of cell plating affords the advantage of a low-resolution requirement for intra-cell analysis, making image-based cell profiling a valuable technique in scenarios where instrument limitations are present [21].

The tissue microenvironment contains richer biological information, which helps to study diseases more intuitively. Hence, it is meaningful to image a whole tissue section at a single-cell scale, which maintains the relationships of intercellular interactions in the tissue microenvironment. For whole tissue sections, the steps of sample preparation are the same as traditional MSI. Samples were obtained by cutting ~10 μm sections from frozen tissue or paraffin-embedded (FFPE) tissue. A high resolution MSI technique is the cornerstone of cell imaging for whole tissue sections, as the cells are crowded on the sections and too large a pixel may contain multiple cells.

### 3.2. Recent Developments in High Spatial Resolution Instruments

The average cell size ranges from 20 to 30 μm for organisms such as live cells and skin cells, with exceptions such as microglia, whose typical diameter measures 7 to 12 μm. The size of cellular organelles is even more diminutive, with structures like the endoplasmic reticulum and Golgi apparatus measuring less than 1 μm in diameter. Consequently, high spatial resolution mass spectrometry imaging (MSI) instruments are required to investigate the lipidome at a cellular or subcellular level. Microscope mode MSI utilizes a broad laser beam to irradiate the sample in a manner similar to that of a conventional microscope light source. The simultaneous acquisition and preservation of the distribution pattern of specific ions during ionization is a distinguishing aspect of this method, enabling resolution without the limitations imposed by ionization techniques [24,25]. Additionally, the flight path of the ion is monitored to maintain this distribution pattern. In a recent study, microscope mode MSI was used to image the stimulus-induced production of endocannabinoids in single neurons at the subcellular level [26]. However, a comprehensive analysis of microscopy mode MSI is not included in this review because its performance at the mass spectrometry level is poor, with lower mass resolution, which has limited its widespread acceptance and application. On the other hand, conventional microprobe mode MSI employs a scanning probe to determine the location of molecules on the sample surface. For microprobe mode MSI, sample surface analysis ions can be generated by laser ablation, ion beam bombardment, and droplet extraction, followed by mass spectrometry analysis, and the lateral spatial resolution of the ion image being restricted by the diameter of the scanning laser (MALDI), ion beams (SIMS) or droplets (DESI). For MSI in microprobe mode, the ion beam can be most easily focused, reaching a spatial resolution as high as tens of nanometers. Laser ablation based MSI methods can reach a resolution of several micrometers, followed by that of DESI in the tens of micrometers range. Given this, laser-based MSI and ion-beam-based MSI are currently the most widely used methods for cellular and subcellular imaging. In this discussion, we will delve into the principles and recent advancements in these two MSI techniques.

#### 3.2.1. Laser-Based MSI

**Figure 3:**
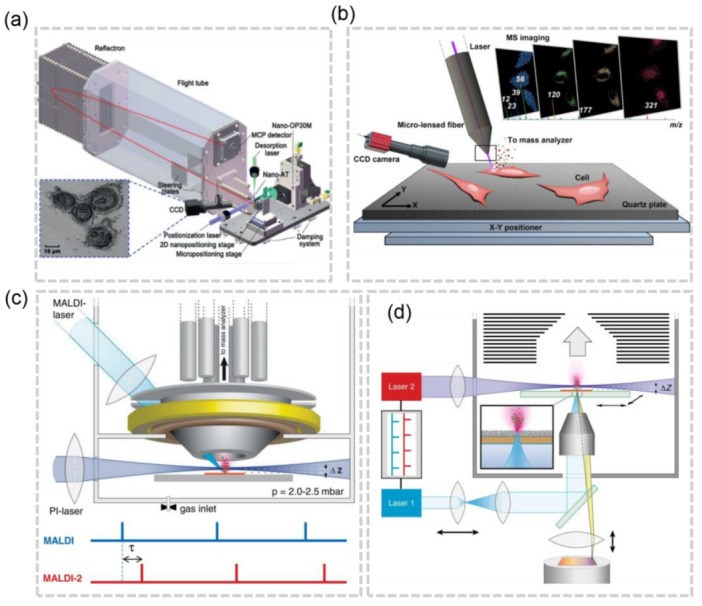
(**a**) Schematic of the NDPI–TOFMS system [28]. (**b**) Schematic of the micro–lensed fiber LDI-MS system [29]. (**c**) Schematics of MALDI–2–MSI [30]. (**d**) Schematics of t–MALDI–2–MSI [22].

The resolution of laser-based MSI can vary based on the type of laser focusing used. There are two main categories: far-field technology and near-field technology. Near-field methods involve pixel ablation at distances from the sample surface that are much smaller than one wavelength (typically in the nanoscale range), while far-field methods ablate pixels at distances larger than this value (typically in the millimeter or centimeter scale). Far-field technology has its resolution limited by optical diffraction, whereas near-field technology does not have this limitation. Within near-field technology, there are further subcategories including apertureless tip enhancement techniques and aperture tip desorption techniques [27]. The group led by Wei Hang has made significant contributions to the advancement of near-field-based MSI with aperture tip desorption. In 2019, Hang et al. developed a near-field desorption post ionization time-of-flight mass spectrometer (NDPI-TOFMS) which was able to achieve a resolution ranging from 250 nm to 350 nm, and even imaged HeLa cells with a resolution of 250 nm(Figure 3a) [28]. They continued to innovate by introducing Micro-Lensed Fiber Laser Desorption Mass Spectrometry Imaging, allowing for cell imaging at a resolution of 300 nm without the need for an AFM imaging process, which can often be cumbersome (Figure 3b) [29].

Matrix-assisted laser desorption/ionization (MALDI) is a most widely used ionization technique of far-field-based MSI [31]. The fundamental principle behind MALDI is the ionization of the sample through laser-assisted desorption and ionization. To achieve this, a sample is combined with a matrix, which absorbs the laser energy and facilitates the desorption and ionization of the sample molecules. The resulting sample-matrix mixture is then deposited on a sample target and subjected to a laser pulse. This laser pulse causes the sample molecules to be ionized and desorbed from the matrix into the mass spectrometer for analysis. For the applications of MALDI in single-cell imaging, spatial resolution is the most important limiting factor. The spatial resolution of MALDI is influenced by several factors, including laser spot size, matrix size, and sample preparation method. The laser spot size is a crucial factor in determining the spatial resolution of MALDI, as smaller laser spots provide higher spatial resolution. However, this comes with the disadvantage of lower sample collection efficiency and signal intensity. The size of the matrix crystals used in MALDI also contributes to the spatial resolution, with larger crystals scattering the laser light and reducing the spatial resolution [32]. Finally, the sample preparation method used can impact the spatial resolution of MALDI, with smaller sample spots deposited onto the MALDI target, resulting in higher spatial resolution, while larger spots scatter the laser light and reduce the spatial resolution.

For most commercial instruments, the pixel size of MALDI MSI are in the range of 5–20 μm [33], which is not adequate for imaging at the cellular or subcellular level. Theoretically, a higher resolution can be achieved by focusing the laser beam to a smaller pixel. However, reduced ion abundance and ion suppression effects can hinder the acquisition of an ion image of high quality at high spatial resolution. The post-ionization (PI) strategy, laser-induced post-ionization (MALDI-2), in which the beam of a PI laser interacts with the expanding particle plume in an nitrogen cooling gas, has been developed to increase the ion yields at a ~5 μm lateral resolution [30] (Figure 3c). By improving the laser focusing objective and applying the matrix, the atmospheric pressure (AP) MALDI setup with a lateral resolution of 1.4 μm has been used to identify various metabolites expressed at the subcellular scale in a single-celled eukaryotic organism *P. caudatum* [34]. To elevate the MALDI-MSI to a new level, M. Niehaus et al. proposed the t-MALDI-2 [22], which combines the transmission-mode MALDI with MALDI-2 (Figure 3d). In t-MALDI-2, the 600 nm resolution has been achieved and the distribution of glycolipids and phospho- in a Vero B4 cell has been visualized. With ongoing advancements in the technology and a better understanding of the factors that influence spatial resolution, such as laser focusing objectives and matrix applications, it is expected that laser-based MSI will continue to evolve and provide even more precise and detailed results.

#### 3.2.2. Ion-Beam-Based MSI

As the most widely used ion-beam-based MSI, Secondary-ion mass spectrometry (SIMS) is a surface analysis technique that utilizes a focused primary ion beam to sputter surface molecules from a sample and create secondary ions [35]. The secondary ions are then analyzed by a mass spectrometer to determine their mass-to-charge ratios. SIMS provides high-resolution, subsurface chemical imaging with lateral resolutions in the nanometer range, which allows researchers to identify chemical compounds at the molecular level within complex samples, providing a detailed understanding of their composition and distribution. In the realm of single-cell imaging, the detection sensitivity of secondary ion mass spectrometry (SIMS) and the minimization of ion fragmentation are of utmost importance. One of the inherent limitations of SIMS is the limited abundance of molecular ions available for lipid structure identification due to the generation of a large number of fragment ions. The size of the primary ion beam has a direct effect on the spatial resolution, with smaller beam sizes providing improved resolution but also reducing the number of ions. This presents a challenge in lipid annotation, as the number of molecular ions becomes even more limited. Furthermore, the energy of the primary ions has a significant impact as it determines the depth of sample penetration. While using higher energy ions can result in a smaller primary ion beam size, it also increases fragmentation and complicates molecular annotation efforts.

**Figure 4:**
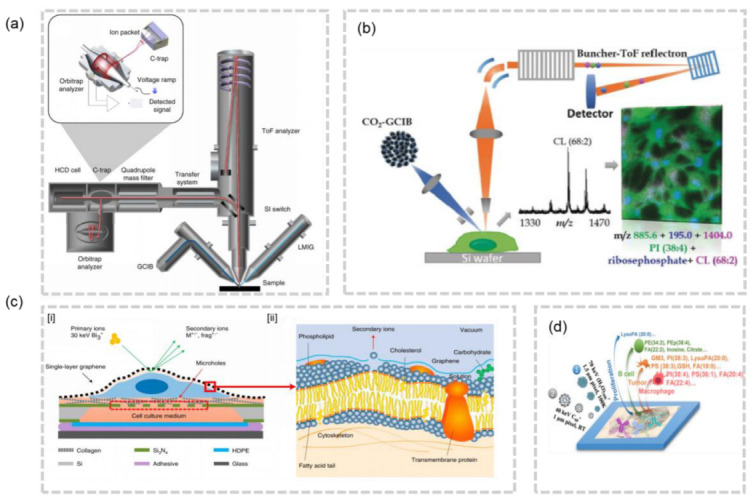
(**a**) Schematics of 3D OrbiSIMS [36]. (**b**) Schematics of a high-energy GCIB-SIMS MSI with Lateral Resolution of 1 μm [37]. (**c**) [**i**] Schematics of the SIMS imaging analysis based on single-layer graphene. [**ii**] The sputtering process for the head group of a phospholipid molecule from a wet cell membrane [42].(**d**) Schematic of the workflow on the cell-type specific profiling of multiomics [41].

SIMS can achieve a lateral resolution of 10 nm in specific configurations. In order to effectively gather enough signals from secondary ions produced by biological samples such as lipids, a lateral resolution of −100 nm is often utilized. This resolution is deemed adequate for imaging cellular structures, but the limitations of hard ionization mode and low ion yield in small pixels have restricted the full potential of SIMS for cellular/subcellular lipidomics analysis. Recently, researchers have made strides in addressing these limitations by both increasing the ion yield and softening SIMS. Gas Cluster Ion Beam SIMS (GCIB-SIMS), an ionization method that minimizes fragmentation, has been developed as an alternative to conventional SIMS. The development of 3D OrbiSIMS, a hybrid instrument that combines the high spatial resolution of GCIB-SIMS and the high mass resolution of orbitrap, has enabled the visualization of metabolites in three dimensions with subcellular resolution [36] (Figure 4a). The type of cluster ion beam used in GCIB-SIMS has a significant impact on the imaging quality. To further improve the resolution of GCIB-SIMS, the Winograd group proposed the use of a 70 keV CO_2_ cluster ion beam. This innovative approach has been shown to effectively image high molecular weight phospholipids (PLs) in subcellular structures at a resolution of 1 μm [37] (Figure 4b). Furthermore, the John C. Vickerman group discovered that the use of H_2_O clusters eliminates the compromise between sensitivity and resolution, resulting in a 10–100 times increase in ion production compared to Ar_1000_ beams and C_60_ [38]. When compared to Ar_2000_ cluster beams, water cluster beams have the potential to enhance the ion yield in a 1 μm^2^ area by 100–1000 times [39]. This innovative H_2_O cluster GCIB-SIMS has been successfully applied to image neurons in frozen-hydrated samples at the single-cell or subcellular level [40]. In addition, SIMS has low sample surface damage, allowing for multiple analyses or coupling with other imaging modalities. For example, (H_2_O)n-GCIB and C60-SIMS can be combined to enable the multi-omics imaging of different cell types in breast cancer tissues (Figure 4d) [41]. It is also worth noting that Dae Won Moon et al. achieved the subcellular SIMS imaging of wet cells without treatment by using single-layer graphene, preserving the natural distribution of lipid molecules in cells to the greatest extent possible (Figure 4c) [42]. These advances in SIMS-MSI have opened new avenues for imaging biological samples with unprecedented detail and clarity.

### 3.3. Data Acquisition of Single Cells and Organelles

Although the mass spectra are recorded at a single-cell scale, the MSI data are not sufficient for the single-cell analysis directly. This is because the correlation between the spectra and the cells cannot be established, and there is no visual representation of the cell size, shape, and location through MSI. Moreover, the cell membranes of the majority of cells on the tissue sections or on the Petri dishes are tightly adhered to each other, with thicknesses ranging from 7–8 nm [43], meaning that a pixel may contain the lipid analytes from two or more cells, even if the resolution is ultra-high. Furthermore, tissue sections are typically cut from fresh frozen or paraffin-embedded tissues, and an unfavorable cutting angle and speed could result in cell stacking. In light of these difficulties, single-cell segmentation methods have been introduced to accurately assign the measured data to individual cells for subsequent analysis. Cell segmentation is supposed to detect the cells and cut the counter of each cell precisely, aiming to label each pixel with the corresponding cell.

In the past decades, the techniques for single-cell recognition are gradually maturing, especially as unsupervised and deep learning methods in medical imaging are on the rise. U-Net [44,45] is a pioneering algorithm to apply segmentation to single-cell analysis via deep neural networks, which generate pixel-level annotations of cell edges, cell interiors, and backgrounds. These annotations are then transformed into the final segmentation mask via thresholding of the probability maps. Another kind of segmentation approaches involve the initial localization of individual cells through the employment of a rough representation of shape, followed by a subsequent refinement of said shape. The bounding boxes that are used as means of localization can be subjected to a process of refinement, resulting in an instance segmentation, which is achieved through the classification of the pixels contained within each box. A notable example of this methodology can be seen in the implementation of Mask R-CNN [46]. However, this kind of strategy is not suitable for a situation where the cells are crowded (e.g., on a tissue section). U-Net plays an important part in the field of biological image segmentation, because many outstanding works are conducted on the basis of the U-Net. On the basis of U-Net, Stardist [47] predicted the a star-convex polygon for each pixel, while Cellpose [48,49] was proposed as a robust algorithm for cellular segmentation, and employs the vertical and horizontal gradients of the topological maps of cells and significantly outperforms Mask R-CNN and Stardist. In a recent study, Noah F. Greenwald et al. constructed Mesmer, which represents a singular, user-friendly solution for cell segmentation that achieves human-level accuracy across various tissue types and imaging techniques, without requiring manual parameter adjustments from the user [50].

**Figure 5:**
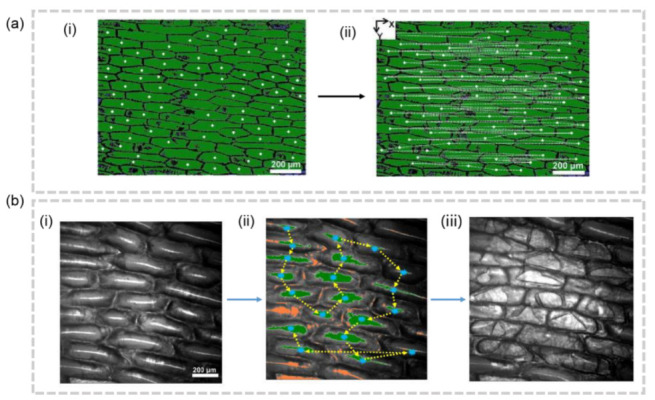
(**a**) Coordinate-guided cellular MSI [51]. (**i**) The centroid of cells are found on the microscope images. (**ii**) The movement of X-Y stage is shown by the dash line. (**b**) Validation for the centroid-based cellular MSI. Images under optical microscope before sampling (**i**), were sampled by predetermined trajectory (**ii**), and after sampling (**iii**).

Despite significant progress in the development of segmentation techniques, accurately assigning measurement data to individual cells remains a challenge in the field of single cell analysis. Two main methodologies exist to tackle this issue. The first method involves segmenting the cells first and then using the segmentation results to guide the sampling process. On the other hand, the second method involves conducting the sampling according to conventional techniques, followed by data assignment. Akos Vertes et al. tried to segment the cells first, and then find cell centroid positions, enabling the automation of sequential f-LAESI-MS analysis of tissue-embedded single cells through programmable automatic sampling on L.longiflorum leaf (Figure 5a) and Allium cepa bulb (Figure 5b) [51,52]. However, this approach has only been validated for plant tissues so far, likely due to the larger size and clearly visible cell walls of plant cells under light microscopy, which makes cell segmentation achievable through the thresholding of gray-scale levels.

**Figure 6:**
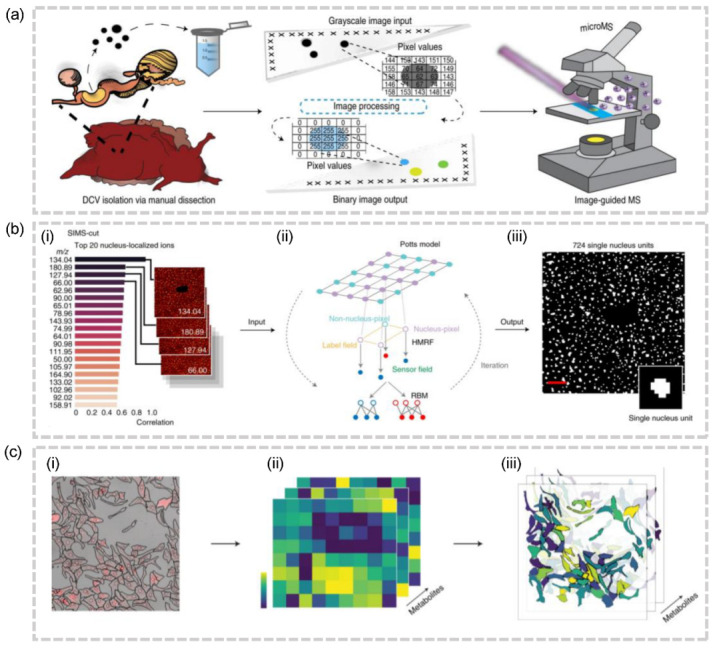
(**a**) Schematic of image-guided MALDI-MSI workflow for high-throughput single-DCV measurements [53]. (**b**) The schematic of the cell segmentation(SIMS-cut) used in SEAM [23]. (**i**) The top 20 ions localized in the nucleus. (**ii**) The utilization of the Potts model as a prior for pixel labels and the use of restricted Boltzmann machines (RBMs) as a conditional distribution for pixel intensities. (**iii**) The resulting nucleus segmentation mask. (**c**)The schematic of SpaceM. Alignment of the segmented microscope image (**i**) with the MALDI-MSI images (**ii**) to obtain the integrated image (**iii**).

Although the feasibility of these protocols in animal tissues has yet to be conclusively proven, it is feasible to apply similar ideas to the imaging of individual organelles. Jonathan V. Sweedler and colleagues proposed image-guided protocols that leverage brightfield images and sequences of image algorithms to automatically identify the location of each dense-core vesicle (DCV) and electron-lucent vesicle (LV) and profile their peptide and lipid contents (Figure 6a) [53]. In 2021, spatial single nuclear metabolomics (SEAM) was introduced as a means to achieve the spatiotemporal analysis of metabolomics at a single-nuclear scale. This methodology relies on the segmentation of regions of interest, accomplished through the use of metabolic markers, such as *m*/*z* 134.04 as a nuclear [54] marker, and is capable of accommodating a diverse range of biological samples, from cell culture assays to complex tissue sections (Figure 6b) [23]. The task of segmenting nuclei is relatively straightforward, as they can be detected by means of their genetic material, metabolite composition, and optical properties. Besides, the scattered distribution of nuclei within the tissue, in contrast to the compact distribution of cells, presents a convenience for this methodology. Despite its scalability and ability to segment nuclear regions, SEAM still requires prior knowledge of the corresponding metabolic markers, and the assignment of lipid content in the cytoplasm, organelles, and cell membrane remains elusive. Aligning the segmented microscopy images before laser ablation with the post-MALDI microscopy images which record the laser ablation marks, SpaceM assigns the individual ablation marks (i.e., pixels) to cells (Figure 6c) [54]. This method has no data redundancy in that every pixel is labeled with the corresponding cells and can be used for subsequent analysis. SpaceM reveals the metabolic states of cultured cells, but its applicability to tissue-embedded imaging has not been evaluated.

## 4. Limitations and Future Perspectives

### 4.1. High-Throughput

The utilization of image-based analysis strategies is commonly known for its high-throughput nature. However, in the context of Mass Spectrometry Imaging (MSI), the imaging process for the analysis of the lipidome of individual cells on a pixel-by-pixel basis can be rather lengthy. This is particularly true for microprobe-mode MSI, where higher resolution results in a prolonged imaging process and the requirement for ultra-high resolution when imaging at the cellular/subcellular level. Moreover, the implementation of tandem mass spectrometry further limits imaging throughput. Efforts have been directed towards accelerating high-resolution MSI, such as the implementation of compressed sensing, and dynamic sparse sampling [55,56], with the aim of improving throughput. However, the imaging process still requires the comprehensive scanning of all pixels on the slides, even though only a fraction of these pixels correspond to the cells or organelles of interest. To overcome this limitation and enhance MSI throughput, it is imperative to first precisely locate each cell or organelle, that is, to perform single-cell or organelle recognition, and then to program the scan path accordingly. Historically, this task was achieved through manual means, which was both time-consuming and labor-intensive. To address this issue, various statistical and supervised learning techniques, such as support vector machines, genetic algorithms, and Markovmodels [57,58,59], have been employed to automate single-cell or organelle recognition. Moreover, the utilization of color information in cell images, in addition to the conventional grayscale changes at cell edges, has been shown to be an effective means of assisting cell detection [60], but only in imaging techniques that capture information in color dimensions. With the advent of deep learning, various protocols have been established that leverage deep neural networks to increase the efficiency and accuracy of cell detection [61,62,63,64]. In conclusion, the low throughput of single-cell MSI can pose a significant limitation to its widespread use, and there remains ample room for improvement in this area. The integration of advanced techniques such as deep learning and image-guided protocols may hold the key to resolving this issue and advancing the field of single-cell imaging.

### 4.2. Sensitivity and Coverage

Sensitivity refers to the ability to detect low levels of analytes within the sample and is often quantified by the limit of detection (LOD). High sensitivity is of utmost importance in high spatial resolution mass spectrometric imaging (MSI) due to its ability to detect molecular species with low abundance within cells and subcellular structures. This attribute is particularly relevant in biological systems where the molecular composition of cells and subcellular structures can exhibit significant variability, and where low-abundance molecular species can have a critical impact on cellular processes. The specific roles played by different isomers of phosphatidylcholine (PC) in cell signaling and membrane organization have been well documented in the literature. These lipid isomers can be distinguished through tandem mass spectrometry. However, the high spatial resolution of the instrument poses a challenge in terms of the abundance of ions that can be analyzed in a single pixel during MS analysis. The higher the resolution, the fewer ions that are available for analysis, making it difficult to perform multiple MS/MS analyses. The success of wide-coverage MS/MS analysis at the single-cell scale heavily relies on the sensitivity of the instrument. This is because the sensitivity of the instrument determines the minimum amount of sample that can be analyzed in a single pixel, as well as the accuracy and reliability of the results. A more sensitive instrument can detect low-abundance species, improving the coverage of lipid structure identification and enabling a more comprehensive analysis of cell signaling and membrane organization. In the context of single-cell or organelle analysis, sensitivity and the low abundance of molecules can significantly limit the variety of lipids that can be structurally identified. However, merely increasing sensitivity is not enough, as traditional data-dependent acquisition (DDA) tandem mass spectrometry analyzes only a few ions within a narrow isolation window at a time to identify their structures, while other analytes are wasted and can only be analyzed in subsequent injections. The development of data-independent acquisition (DIA) tandem mass spectrometry methods has increased the utilization rate of analytes with lower abundance to some extent, as multiple ions can be fragmented concurrently. However, the obstacle to the application of DIA lies in how to analyze the obtained mass spectra, and various deconvolution methods have been proposed to make it possible to use DIA mode MSI for the analysis of lipid spatial distribution [65,66]. In a study at a tissue level, Guo et al. utilized photochemical derivatization to enhance the imaging of 20 phospholipid C=C-localized isomers [67]. This method has the potential to achieve single-cell scale identification of the spatial location of these lipid isomers, as long as the sensitivity of the imaging is improved to an adequate level. Therefore, the optimization of sensitivity remains a lively area of research, with ongoing efforts aimed at developing advanced techniques to improve the LOD and enhance the accuracy of MSI experiments. Moreover, existing research has demonstrated that the sensitivity of mass spectrometry imaging can be improved through chemical derivatization methods that enhance ionization and increase molecular stability [68]. The development of these techniques is crucial for the advancement of MSI in the field of biological research, allowing for a more precise and comprehensive analysis of cellular and subcellular processes.

### 4.3. Multimodal Imaging

The integration of MSI with other imaging modalities offers a more comprehensive insight into cellular information, especially when MSI alone is not yet capable of providing sufficient detail for single-cell imaging. As previously mentioned, MSI faces difficulties in characterizing the size, shape, and boundaries of cells in tissue sections, which can be easily achieved through other imaging techniques such as fluorescence microscopy. However, the implementation of multimodal imaging presents several challenges, including the requirement for the preservation of the molecular composition of cells and the avoidance of significant ion suppression. The integration of multiple sets of data generated from different imaging modalities requires accurate alignment at the micron level, given the micron-scale resolution of cellular structures.

The integration of multiple ionization methods of MSI has the potential to yield a more comprehensive analysis of substances. While each single ionization technique has its limitations, the combination of multiple MSI modes provides a broader scope of substance analysis. For instance, MALDI excels in detecting large molecules such as proteins, however it lacks the necessary spatial resolution. Conversely, SIMS boasts a high spatial resolution, yet the molecular ions produced are low in abundance and biased towards smaller molecules like metabolites. By integrating MALDI-MSI with SIMS-MSI, researchers have been able to enhance the information content and image quality of mass spectrometry imaging [69,70]. Furthermore, even within the same class of ionization imaging, multiple modalities can be integrated, as demonstrated by the successful combination of H_2_O cluster GCIB-SIMS and C_60_ SIMS. The multimodal MSI offers the potential to provide a more comprehensive understanding of cellular biology. However, it also presents a number of challenges that must be addressed in order to obtain accurate and reliable results. The development of robust and reliable multimodal imaging methods will significantly enhance our ability to study cellular biology at a high level of detail.

## 5. Conclusions

In recent years, advancements in mass spectrometry imaging (MSI) of high spatial resolution and sensitivity have paved the way for analysis in cellular/subcellular lipidomics. With improved techniques and methods for data analysis, MSI is now a powerful tool for complete cellular lipidomics analysis. By providing in-depth information on the spatial distribution and structure of lipids in individual cells, MSI has the potential to greatly deepen our understanding of metabolic diversity within tissues and the crucial role of lipids in regulating cellular behavior. The detailed characterization of biomolecules from single cells or organelles in tissues also requires tandem MS imaging, which may become the next climax of MSI. Certainly, instrumental and methodological innovation are necessary to support such research efforts. However, limitations such as the speed and sensitivity of MSI imaging, as well as the challenge of assigning measurement data to individual cells, may present barriers to the widespread application of MSI in cellular lipidomics. Nevertheless, these limitations also present opportunities for future development and improvement in the field, particularly in the development of computational methods [71]. The integration of multimodal imaging methods has the potential to bring a fresh perspective to cellular lipidomics and further expand our knowledge of lipid metabolism and its impact on cellular behavior. It is worth mentioning that functional MSI (fMSI) represents a new and widely applicable in situ bioactive imaging method, and the progress of many fMSI-related works also indicates that there may be more possibilities for single-cell functional lipidomics analysis through mass spectrometry imaging [72]. In conclusion, as we continue to stay updated on the latest advancements in the field, we can expect to see more breakthroughs and exciting developments in cellular/subcellular lipidomics through the application of MSI technology.

## Data Availability

No new data were created or analyzed in this study. Data sharing is not applicable to this article.
